# The Emergence of Psilocybin in Psychiatry and Neuroscience

**DOI:** 10.3390/ph18040555

**Published:** 2025-04-09

**Authors:** Hossein Omidian, Alborz Omidian

**Affiliations:** 1 Barry and Judy Silverman College of Pharmacy, Nova Southeastern University, Fort Lauderdale, FL 33328, USA; 2 Department of Psychiatry, Westchester Medical Center, Valhalla, NY 10595, USA; alborz.omidian@wmchealth.org

**Keywords:** psilocybin, clinical trials, neuroplasticity, psychiatric disorders, psychedelic therapy

## Abstract

Psilocybin, a naturally occurring psychedelic compound, has garnered renewed scientific interest for its potential in treating psychiatric and neurological disorders. This review systematically examines the latest research on psilocybin’s pharmacokinetics, pharmacodynamics, clinical efficacy, and safety profile. Emerging evidence supports its efficacy in conditions such as major depressive disorder (MDD), treatment-resistant depression (TRD), anxiety, alcohol use disorders (AUD), and cancer-related distress. Despite promising outcomes, significant barriers remain, including methodological constraints, regulatory hurdles, and limited population diversity in clinical trials. Advances in biosynthetic production and optimized psychotherapeutic integration are necessary to ensure scalability and accessibility. Future research should focus on long-term safety, dosing precision, and neurobiological mechanisms to refine its therapeutic applications. This review provides a critical foundation for advancing evidence-based clinical integration of psilocybin.

## 1. Introduction

Psilocybin, a naturally occurring tryptamine alkaloid found in certain mushroom species, has re-emerged in psychiatric research due to its potential therapeutic effects. As a prodrug, psilocybin is rapidly converted into psilocin, which acts as a partial agonist at serotonin 5-HT_2_A receptors—an interaction central to its influence on perception, cognition, and affective processing [1]. This serotonergic activity underpins a range of downstream effects, including altered brain connectivity and emotional modulation.

Recent neuroimaging studies have demonstrated that psilocybin increases functional integration across brain networks, particularly within higher-order cognitive systems. These connectivity changes have been associated with reductions in depressive symptoms, suggesting a neurobiological basis for psilocybin’s therapeutic potential in MDD and related conditions [2]. Additionally, region-specific changes in glutamatergic signaling—particularly in medial prefrontal and hippocampal regions—further implicate psilocybin in the modulation of neural circuits involved in emotional regulation and self-referential processing [2,3].

While mechanistic studies provide insight into how psilocybin acts on the brain, clinical trials have demonstrated its efficacy in reducing symptoms of depression and alcohol dependence. A systematic review of clinical studies reported significant antidepressant effects across multiple trials [4], and randomized controlled data indicate reductions in heavy drinking days among individuals receiving psilocybin-assisted therapy for AUD [5]. Although the precise biological mechanisms are still under investigation, these clinical outcomes underscore psilocybin’s therapeutic relevance for mood and substance use disorders.

Beyond its current medical applications, psilocybin has a long history of use in Indigenous spiritual and healing practices. Modern clinical research, however, is often conducted in controlled, Western medical environments with limited acknowledgment of these cultural contexts. Recent bibliometric analyses reveal a growing disconnect between traditional practices and contemporary clinical methodologies, raising important ethical considerations for equitable and culturally informed policy development [6].

In terms of safety, psilocybin has shown a favorable profile in controlled settings. Meta-analyses of clinical trials report low cardiotoxicity and minimal impact on QTc intervals, indicating limited cardiovascular risk [7,8]. However, certain populations—such as individuals with bipolar disorder or significant trauma histories—may require additional risk stratification due to the potential for psychological destabilization [1,9,10].

Psilocybin’s resurgence in biomedical research has also spurred technological and methodological advancements. Innovations in biosynthetic and hybrid enzymatic production have enhanced scalability for pharmaceutical development [11], while psychotherapeutic integration models such as Acceptance and Commitment Therapy (ACT) and Compassion-Focused Therapy (CFT) are being explored to optimize treatment outcomes. Nonetheless, challenges remain in standardizing dosing protocols, ensuring long-term follow-up, and refining therapeutic frameworks to maximize both efficacy and safety.

This review synthesizes current evidence from clinical trials and mechanistic studies, critically examining psilocybin’s psychiatric applications, production innovations, and ethical considerations. It also identifies research gaps and methodological barriers, offering a comprehensive overview of psilocybin’s evolving role in psychiatry and neuroscience.

## 2. Overview of Psilocybin Research Categories

To contextualize the current landscape of psilocybin research, Table 1 categorizes existing studies into four major domains: systematic reviews, clinical trials, neuroimaging studies, and special population investigations. This categorization highlights the breadth of evidence supporting psilocybin’s psychiatric potential, while also identifying methodological gaps and safety considerations.

Systematic reviews synthesize findings across multiple studies, offering high-level insights into psilocybin’s therapeutic effects [4,9,10,12,13,14,15,16,17]. These reviews generally support the compound’s potential for reducing symptoms of depression, anxiety, and alcohol use disorder (AUD). However, they also emphasize limitations such as small sample sizes, variability in dosing and protocols, expectancy effects, and the impact of regulatory barriers. Mild to moderate adverse effects are commonly reported, while rare but serious adverse events underscore the need for further investigation and caution in clinical adoption.

Clinical trials, including randomized controlled studies, have demonstrated significant symptom reductions in major depressive disorder (MDD), treatment-resistant depression (TRD), and AUD [5,18,19,20,21,22,23,24,25]. Some findings suggest therapeutic outcomes comparable to standard antidepressants. Nonetheless, outcomes vary across individuals, and expectancy effects may influence perceived efficacy. While short-term adverse effects are frequently observed, long-term safety data remain limited, emphasizing the need for additional well-powered trials to establish optimal treatment protocols and safety benchmarks.

Special population investigations explore psilocybin-assisted therapy in groups with unique medical or psychological needs, such as individuals with terminal cancer, military veterans with PTSD, and patients with complex psychiatric or neurological disorders [26,27,28,29,30,31,32]. Preliminary evidence suggests therapeutic benefits for conditions including cancer-related depression, anorexia nervosa, and chronic cluster headaches. However, these populations often carry elevated psychological risk profiles, and individualized treatment protocols are essential. Larger, rigorously controlled studies are needed to validate efficacy and ensure safety in these vulnerable groups.

## 3. Mechanisms and Translational Potential

### 3.1. Pharmacology and Neuroreceptor Systems

#### 3.1.1. Pharmacokinetics and Dosing

Understanding psilocybin’s pharmacokinetics is critical for establishing effective and safe dosing strategies in clinical settings. In a controlled study involving healthy participants, escalating oral doses (0.3, 0.45, and 0.6 mg/kg) produced linear pharmacokinetics of psilocin, psilocybin’s active metabolite. Psilocin exhibited a mean elimination half-life of approximately three hours, with minimal renal clearance, suggesting no significant need for dose adjustments in individuals with mild-to-moderate renal impairment. No serious adverse events were reported, supporting the compound’s tolerability under clinical supervision [1].

In a complementary study using fixed-dose administration (15 mg, 25 mg, and 30 mg), psilocin plasma concentrations peaked approximately two hours post-ingestion and followed dose-proportional kinetics. The subjective effects of psilocybin lasted between 5.5 and 6.4 h and increased with higher doses. Notably, body weight did not significantly influence pharmacokinetic parameters or subjective responses, providing a rationale for fixed dosing in future clinical trials [34].

#### 3.1.2. Microdosing Research

Microdosing psilocybin—defined as the regular consumption of sub-perceptual doses—has gained attention for its potential cognitive and emotional benefits. A systematic review of clinical trials, sociological analyses, and anecdotal reports found that although users frequently reported enhanced mood and creativity, controlled studies failed to consistently replicate these effects. This discrepancy was attributed to methodological limitations, including small sample sizes, inadequate blinding, and strong expectancy effects. These findings highlight the need for rigorous, placebo-controlled studies to evaluate the therapeutic and pharmacological validity of microdosing practices [16].

#### 3.1.3. Neuroreceptor Activity

Psilocybin’s psychoactive and therapeutic effects are primarily mediated through partial agonism at serotonin 5-HT_2_A receptors. This receptor interaction plays a key role in altering perception, cognition, and emotional processing [1]. Additionally, psilocybin is believed to influence other neurotransmitter systems, notably glutamatergic signaling, which is implicated in cognitive flexibility and neuroplasticity. A double-blind, placebo-controlled neuroimaging study found that psilocybin increased glutamate concentrations in the medial prefrontal cortex (mPFC)—associated with ego dissolution—and decreased levels in the hippocampus, correlating with positive subjective experiences. These findings suggest a dual modulation of serotonergic and glutamatergic pathways, supporting psilocybin’s mechanistic relevance in psychiatric therapy [3].

Beyond serotonergic activity, psilocybin’s broader neuropharmacological profile includes cross-system modulation involving both glutamatergic and dopaminergic signaling. Region-specific alterations in glutamate levels—namely, elevated medial prefrontal and reduced hippocampal glutamate—have been linked to the dual phenomenology of ego dissolution, spanning distressing and positive experiences [3]. These effects parallel increases in cortico-limbic functional connectivity, indicating that neurotransmitter crosstalk may underlie psilocybin’s influence on emotional and cognitive regulation. In preclinical models, psilocybin also increased dopamine D2 receptor expression in the nucleus accumbens and prefrontal cortex of ethanol-conditioned rats, implicating dopaminergic pathways in its potential role in modulating reward and addiction-related behaviors [35].

### 3.2. Brain Networks and Mechanistic Pathways

#### 3.2.1. Functional Brain Connectivity

Psilocybin significantly alters large-scale brain network dynamics. Functional MRI (fMRI) studies demonstrate that psilocybin increases global brain network integration while reducing modularity, thereby enhancing communication between typically segregated networks. In individuals with MDD, these changes have been associated with rapid and sustained symptom improvement. In contrast, treatment with the SSRI escitalopram did not produce similar connectivity changes, suggesting that psilocybin engages distinct neural mechanisms [2].

#### 3.2.2. Cognitive and Emotional Processing

In a randomized trial comparing psilocybin with escitalopram, researchers evaluated the drugs’ effects on cognitive processes linked to depression, including rumination and thought suppression. Psilocybin significantly reduced both maladaptive strategies, with reductions in rumination observed exclusively among clinical responders. These effects were correlated with experiences of ego dissolution and psychological insight, supporting psilocybin’s role in enhancing emotional flexibility through mechanisms distinct from those of conventional antidepressants [36].

#### 3.2.3. Neuroplasticity and Adaptive Learning

Psilocybin’s ability to promote neuroplasticity has been proposed as a key therapeutic mechanism. Pooled neuroimaging data from two clinical trials showed increased functional connectivity within higher-order networks, including the default mode and executive control networks, correlating with reductions in depressive symptoms [2]. Preclinical behavioral research further supports these findings: in a rodent model, psilocybin increased task engagement and reduced loss aversion by altering belief-updating and memory decay parameters, suggesting a role in modulating learning and reward-based behavior [37].

#### 3.2.4. Socio-Emotional Modulation and Therapeutic Connection

In addition to its effects on brain connectivity and cognitive flexibility, psilocybin may influence socio-emotional processing relevant to its therapeutic efficacy. A randomized, placebo-controlled trial found that a single psilocybin dose significantly enhanced emotional empathy toward positive stimuli in individuals with depression, with effects persisting for at least two weeks. This suggests psilocybin may support improvements in social cognition, particularly in disorders marked by social withdrawal or emotional numbing [38].

Psilocybin may also strengthen the therapeutic alliance during psychedelic-assisted psychotherapy. A feasibility study analyzed video recordings from four psilocybin sessions and identified 372 distinct moments of therapist–patient connection, 83% of which were independently verified by multiple coders. Both verbal and nonverbal cues of interpersonal connection varied in parallel with the subjective intensity of the psychedelic experience. These results highlight psilocybin’s potential to enhance psychotherapeutic outcomes by deepening relational engagement and underscore the feasibility of systematically measuring therapeutic dynamics in psychedelic contexts [39].

#### 3.2.5. Behavioral Neuroscience Perspective

Psilocybin appears to enhance cognitive flexibility and emotional regulation, likely via increased neuroplasticity. In animal models, psilocybin augments functional connectivity across cortico-striatal regions and upregulates immediate early genes like EGR1, suggesting widespread cortical activation [40]. Human neuroimaging studies reveal decreased default mode network (DMN) connectivity, associated with disrupted maladaptive self-referential processing [41,42]. These changes correspond with improved emotional empathy [38], reduced anhedonia [43], and enduring shifts in personality domains such as impulsiveness and neuroticism [44]. Together, these findings align with a growing body of evidence implicating psilocybin’s modulation of hierarchical brain networks as a mechanism for long-term behavioral adaptation, cognitive reframing, and psychological resilience.

### 3.3. Protocol Optimization and Predictive Tools

#### 3.3.1. Clinical Trial Innovations

Efforts to refine psilocybin therapy have led to innovations in clinical trial design. A randomized, waitlist-controlled study is evaluating psilocybin’s feasibility and safety in individuals with treatment-resistant obsessive-compulsive disorder (OCD). Participants receive either 25 mg or 30 mg doses with non-directive psychological support, and symptom changes are assessed using the Yale-Brown Obsessive-Compulsive Scale (Y-BOCS). This trial aims to inform future treatment frameworks for anxiety-spectrum conditions [45].

#### 3.3.2. Therapy Personalization Using Predictive Models

Advancements in artificial intelligence have introduced new tools for optimizing psilocybin therapy. In a recent study, natural language processing (NLP) was applied to post-treatment therapy session transcripts from patients with TRD. Sentiment analysis predicted long-term treatment outcomes with an accuracy of 85–88%. These findings suggest that machine learning may be used to identify key linguistic or emotional patterns associated with positive therapeutic responses, facilitating personalized treatment approaches [46].

Table 2 summarizes psilocybin’s pharmacokinetics, pharmacodynamics, and neurobiological effects, highlighting how the substance is metabolized, interacts with the brain, and influences neural connectivity. Pharmacokinetic studies show psilocybin is rapidly converted to psilocin, with a half-life of approximately 3 h and minimal renal clearance. Pharmacodynamic research reveals psilocybin primarily acts as a 5-HT2A receptor agonist, modulating serotonin levels and influencing cognition, mood, and perception. Neuroimaging studies indicate psilocybin reduces default mode network (DMN) activity, increases brain network integration, and may promote neuroplasticity. While findings suggest potential therapeutic applications in mood and psychiatric disorders, long-term structural effects and mechanisms require further investigation.

## 4. Therapeutic Framework and Considerations

### 4.1. The Role of Psychotherapy in Enhancing Psilocybin Outcomes

The integration of psychotherapy with psilocybin administration is increasingly recognized as a critical factor in optimizing clinical outcomes. One placebo-controlled study investigated psychological flexibility as a mediating mechanism in psilocybin therapy for individuals with MDD. The results demonstrated that psilocybin significantly improved psychological flexibility, mindfulness, and values-based living. These psychological improvements strongly correlated with reductions in depression severity. The study concluded that structured psychotherapeutic approaches, such as ACT, which emphasize values-based action and psychological flexibility, may enhance the therapeutic efficacy of psilocybin [49].

In addition to ACT, Compassion-Focused Therapy (CFT) has been proposed as a complementary psychotherapeutic framework for psilocybin-assisted treatment of depression. A structured protocol outlined how CFT could be integrated into both the dosing and integration phases of psilocybin therapy. CFT focuses on developing self-compassion, emotional regulation, and resilience—elements that align with psilocybin’s facilitation of emotional insight and processing. While the model has not yet been empirically tested in large-scale trials, it presents a theoretically grounded approach for augmenting emotional healing during psilocybin therapy sessions [50].

Psychotherapeutic integration is a defining feature of psilocybin therapy, but models vary widely. Structured approaches like ACT [51] and the Compass Psychological Support Model (CPSM) [52] appear to enhance emotional insight and long-term outcomes, particularly when sessions are designed to reinforce psychological flexibility [49]. Studies show that therapeutic alliance strongly predicts depression outcomes and mystical experience intensity [53,54]. A deeper therapeutic bond prior to dosing is associated with greater emotional breakthrough, while alliance post-session predicts long-term clinical improvement [53]. However, trials often underreport psychotherapeutic fidelity, and process evaluations are needed to understand how specific psychotherapeutic elements interact with the psychedelic state [55].

Psilocybin has demonstrated rapid and sustained therapeutic effects across MDD [19,25], TRD [22,43], AUD [44,48,56], BDD [33], and cancer-related anxiety [57,58]. However, systematic comparisons are limited by variability in design and outcome measures. Adverse events, including worsened suicidal ideation [59] and re-emergence of trauma symptoms [60], emphasize the importance of structured screening protocols. Standardized assessments of psychiatric history, trauma exposure, and psychological resilience are critical for risk mitigation, particularly in vulnerable populations.

### 4.2. Safety, Adverse Effects, and Ethical Considerations

When administered in controlled clinical settings, psilocybin is generally associated with a favorable safety profile. A meta-analysis of six randomized clinical trials involving a total of 528 participants reported commonly observed side effects, including nausea, headache, dizziness, transient anxiety, and mild elevations in blood pressure. These adverse effects were typically short-lived and resolved within 48 h without requiring medical intervention. The study emphasized the importance of continued research to refine risk mitigation strategies and improve patient monitoring protocols in broader clinical applications [9].

Another systematic review and meta-analysis, which evaluated psilocybin’s antidepressant efficacy across 10 clinical trials (eight included in the quantitative analysis, totaling 524 patients), confirmed that psilocybin produced significant reductions in depressive symptoms. Higher doses were associated with greater therapeutic benefits. Both short-term and long-term effects were observed. While most adverse effects were transient and reversible, some serious adverse events were reported, underscoring the need for comprehensive safety evaluations in ongoing and future studies [4].

Beyond physical safety, ethical considerations are vital in the development and implementation of psychedelic-assisted therapies. A study specifically examined the implications of ego dissolution—a commonly reported psilocybin experience—in psychiatric treatment contexts. The authors raised concerns about potential personality and worldview changes following psilocybin sessions and emphasized the need for thorough informed consent procedures. To enhance ethical transparency, the study proposed specific discussion prompts to guide clinicians in preparing patients for potential psychological and existential transformations, thereby supporting ethically responsible clinical practice [61].

### 4.3. Public Perception and Access

Public interest in psilocybin’s therapeutic potential is increasing, accompanied by evolving perceptions of its safety and efficacy. A national survey conducted in Norway (N = 1078) assessed psilocybin use and public attitudes toward its clinical application. While only 8% of respondents reported previous psilocybin use, 51% expressed openness to trying it as a medical treatment. These results indicate a relatively low rate of recreational use but a high level of support for clinical exploration, which may facilitate future research initiatives in Scandinavian populations [62].

As psilocybin services gain traction in legal and clinical contexts, efforts are being made to establish standardized therapeutic frameworks. An e-Delphi study recruited 36 international experts in psilocybin therapy to reach a consensus on core process, outcome, and structure measures necessary for safe and effective treatment delivery. The experts identified 11 process measures (e.g., patient preparation), 11 outcome measures (e.g., symptom tracking), and 17 structure measures (e.g., facilitator qualifications and adverse event monitoring). These findings support the development of standardized protocols to guide clinical practice and regulatory compliance in emerging legal psilocybin programs [63].

## 5. Key Insights from Psilocybin Clinical Research

### 5.1. Mood and Anxiety Disorders

#### 5.1.1. Major Depressive Disorder (MDD)

Psilocybin has been increasingly studied for its potential antidepressant effects in individuals diagnosed with MDD. A recent systematic review and meta-analysis of randomized controlled trials synthesized data from 10 studies, 8 of which were included in the meta-analysis, involving a total of 524 adult participants. The pooled results demonstrated a large effect size in favor of psilocybin over comparators (Hedges’ g = −0.89, 95% CI −1.25 to −0.53, *p* < 0.01), indicating a significant reduction in depressive symptoms. The analysis also found that therapeutic effects tended to increase with higher doses of psilocybin. While adverse events were generally transient and reversible, some serious adverse events were reported, underscoring the importance of continued safety evaluations in psilocybin research [4].

A recent multisite, double-blind, phase II randomized clinical trial evaluated the antidepressant effects and safety of a single 25 mg dose of psilocybin in adults with moderate to severe MDD. Compared to an active placebo (niacin), psilocybin was associated with significantly greater reductions in depression severity, as measured by the Montgomery–Åsberg Depression Rating Scale (MADRS), both at 8 days (mean difference = −12.0, 95% CI −16.6 to −7.4) and 43 days (mean difference = −12.3, 95% CI −17.5 to −7.2) post-treatment. Improvements in functional impairment were also observed, and while there were no serious treatment-emergent adverse events, the psilocybin group experienced a higher rate of adverse events overall, including severe events. These results support psilocybin’s potential as a novel treatment for MDD when administered with psychological support [19].

In a comparative study of psilocybin and the SSRI escitalopram, psilocybin was found to significantly reduce rumination and thought suppression—maladaptive cognitive patterns often observed in depression. These reductions were specific to the psilocybin group and were particularly pronounced among treatment responders. Moreover, decreases in these maladaptive processes were correlated with experiences of ego dissolution and session-related psychological insight, suggesting a potential divergence from conventional antidepressant mechanisms [36].

Neuroimaging findings provide additional insights into psilocybin’s mechanisms of action. In two clinical trials, including both an open-label study and a randomized controlled trial, functional MRI data revealed that psilocybin treatment was associated with reduced brain network modularity and increased global integration, particularly within higher-order brain networks enriched with 5-HT_2_A receptors. These changes were associated with sustained reductions in depressive symptoms. In contrast, escitalopram treatment did not produce comparable changes in brain network organization, suggesting that psilocybin may exert its antidepressant effects through a distinct neurobiological mechanism [2].

In addition to these neurobiological mechanisms, psychological processes such as increased psychological flexibility have also been implicated in psilocybin’s therapeutic effects. An exploratory placebo-controlled study found that psilocybin administration led to significant improvements in psychological flexibility, mindfulness, and values-congruent living. These changes were strongly associated with reductions in depression severity over a 16-week period, supporting the integration of psychotherapeutic approaches, such as ACT, alongside psilocybin treatment [49].

Overall, the growing body of clinical research suggests that psilocybin may offer meaningful antidepressant effects in individuals with MDD. However, reports of serious adverse events, while rare, highlight the need for ongoing safety monitoring and the development of standardized protocols for screening, support, and risk mitigation [4].

#### 5.1.2. Treatment-Resistant Depression (TRD)

Psilocybin has been evaluated as a potential intervention for TRD, particularly in individuals who have not responded to conventional pharmacotherapies. In the largest randomized controlled trial of psilocybin to date, 233 participants with TRD received a single dose of COMP360 psilocybin at 25 mg, 10 mg, or 1 mg (control), administered with psychological support. At Week 3, participants in the 25 mg group experienced greater improvements in patient-reported measures of depression severity, anxiety, affect, functioning, and quality of life compared to the 1 mg group. The 10 mg group showed smaller improvements across these outcomes. While the trial demonstrated robust short-term benefits, interpretation is limited by the lack of an active comparator and the possibility of functional unblinding among participants receiving lower doses [20].

A separate open-label phase II study investigated the safety and efficacy of administering a 25 mg dose of COMP360 psilocybin alongside ongoing SSRI treatment in individuals with TRD. Among the 19 participants, significant reductions in Montgomery–Åsberg Depression Rating Scale (MADRS) scores were observed three weeks post-treatment (mean change = −14.9, 95% CI −20.7 to −9.2). Clinical response or remission was achieved in 42.1% of participants. No serious treatment-emergent adverse events were reported, and no indications of increased suicidal ideation or behavior were observed. Most adverse events were mild and resolved on the day of occurrence. These findings suggest that psilocybin may be safely co-administered with SSRIs, although further controlled studies are needed to validate this approach [64].

Qualitative research has also provided insight into patient experiences during psilocybin treatment for TRD. In a study involving 11 participants from a randomized trial, three major themes were identified: (1) challenges with trust-building and managing expectations; (2) navigating the psilocybin experience, including feelings of surrender and the therapeutic role of music; and (3) the need for a more comprehensive and individualized treatment process. Participants emphasized the importance of therapeutic trust, adequate preparation, and the potential value of multiple sessions and post-treatment support. These findings suggest that optimizing the therapeutic context may enhance patient outcomes and support broader real-world applications [65].

A post hoc analysis of data from the large COMP360 trial examined whether discontinuing antidepressant medication prior to psilocybin administration affected treatment outcomes. Among participants who either discontinued antidepressants or were drug-free at study entry, no differences were found in baseline depression severity, suicidality, or treatment efficacy at Week 3. The subjective psychedelic experience was also not significantly impacted by recent antidepressant discontinuation. These results suggest that prior use or discontinuation of antidepressants may not compromise the effectiveness of psilocybin treatment and support its potential use as monotherapy in TRD [66].

#### 5.1.3. Anxiety, Demoralization, and Cancer-Related Distress

Psilocybin-assisted psychotherapy (PAP) has been studied as a potential treatment for anxiety, depression, and existential distress in individuals with serious medical illnesses, particularly cancer. A phase II open-label trial evaluated the safety, feasibility, and preliminary efficacy of psilocybin-assisted group therapy in 30 patients diagnosed with both cancer and MDD. Participants received a single 25 mg dose of psilocybin in a novel group-cohort setting, supported by both individual and group therapeutic preparation and integration. The intervention was associated with a substantial reduction in depression severity scores (mean change: −19.1 points; 95% CI −22.3 to −16.0; *p* < 0.0001) by week 8. Eighty percent of participants demonstrated a sustained antidepressant response, and 50% achieved full remission by week 1, with sustained effects through week 8. No serious adverse events or suicidality were reported, and side effects such as nausea and headache were mild and transient. The study demonstrated that group delivery was both feasible and safe in this population [26].

A meta-analysis combining data from two phase II, randomized, placebo-controlled crossover trials examined the broader psychiatric effects of PAP in cancer patients. Across a sample of 79 participants, PAP significantly improved symptoms across multiple dimensions, including anxiety, depression, interpersonal sensitivity, hostility, obsession-compulsion, and somatization. Importantly, there was no evidence of increased paranoia, phobia, or psychosis, supporting the overall safety of PAP in this population. Clinical improvements were consistent across both trials, indicating the potential of PAP to address a broad spectrum of psychological symptoms in individuals with cancer-related distress [67].

Qualitative and mixed-method studies have explored the subjective experiences of patients undergoing PAP. A report featuring four patient narratives from a double-blind trial of psilocybin for cancer-related anxiety and depression highlighted deeply personal, variable experiences during treatment. Common themes included enhanced self-compassion, acceptance of death, and processing of past trauma. These individualized experiences were associated with improvements in mood, quality of life, and spiritual well-being [68].

In another qualitative study, interviews with 13 participants diagnosed with cancer and clinically elevated anxiety identified ten recurring themes reflecting their experiences with psilocybin therapy. These included confronting mortality, reconciling with the cancer diagnosis, and forming healing narratives. Participants described how the immersive nature of the psychedelic experience led to a deeper emotional detachment from the illness and a renewed connection to life. Music was often noted as a therapeutic guide, and many participants framed their experiences through a spiritual or existential lens. These findings offer insights into the psychological mechanisms underlying psilocybin’s therapeutic effects in cancer-related distress [57].

A randomized controlled trial investigated the long-term effects of PAP on suicidal ideation (SI) and loss of meaning (LoM)—both key features of demoralization and existential distress—in patients with advanced cancer. Psilocybin was associated with rapid and sustained reductions in SI, observable as early as 8 h post-treatment and lasting up to 6.5 months. Significant and lasting reductions in LoM were observed at two weeks and remained evident at follow-ups extending to 3.2 and 4.5 years. These results support psilocybin’s potential as an antisuicidal intervention in cancer patients by alleviating hopelessness and promoting meaning-making, and suggest that it may offer benefits beyond those achievable with traditional antidepressants in this population [58].

### 5.2. Addiction and Impulse-Related Disorders

#### 5.2.1. Alcohol Use Disorders (AUD)

PAP has been investigated as a potential treatment for AUD, a condition associated with high relapse rates and limited long-term treatment success. A double-blind, randomized clinical trial involving 95 participants with AUD assessed the efficacy of psilocybin administered alongside 12 weeks of manualized psychotherapy. Participants received two high-dose psilocybin sessions (25–40 mg/70 kg) or an active placebo (diphenhydramine) at weeks 4 and 8 of the psychotherapy program. Over the 32-week follow-up period, the psilocybin group experienced a significantly lower percentage of heavy drinking days (9.7%) compared to the placebo group (23.6%), with a mean difference of 13.9% (95% CI, 3.0–24.7; *p* = 0.01). Psilocybin was also associated with lower mean daily alcohol consumption. No serious adverse events were reported in the psilocybin group. These findings support the therapeutic potential of psilocybin-assisted therapy for reducing harmful alcohol use [5].

To investigate the mechanisms underlying psilocybin’s effects on alcohol consumption, a preclinical study examined the impact of psilocybin on ethanol self-administration in rats. Psilocybin reduced alcohol intake by approximately 50% when injected systemically or directly into the left nucleus accumbens. This effect was blocked by the administration of a 5-HT_2_A receptor antagonist, suggesting a serotonin receptor-dependent mechanism. In alcohol-consuming rats, psilocybin increased dopamine D2 receptor mRNA expression in the nucleus accumbens and prefrontal cortex, while dopamine D1 receptor expression increased only in the prefrontal cortex. These findings implicate serotonergic and dopaminergic pathways in psilocybin’s modulation of alcohol-related behavior and suggest lateralized brain region specificity [35].

A multi-site, double-blind, randomized controlled trial (N = 96) was conducted to evaluate the effects of PAP on AUD. Participants received two dosing sessions (psilocybin or diphenhydramine) within a structured 12-week therapy platform that integrated motivational enhancement and cognitive-behavioral approaches. Although primary results were not yet published in the referenced article, the study design emphasized the use of an evidence-based psychotherapeutic framework, safety monitoring, and exploration of mediators and moderators of treatment response [56].

A pilot fMRI study further explored the neural effects of psilocybin in individuals with AUD. Eleven participants from the aforementioned clinical trial completed fMRI scans before and after receiving psilocybin or diphenhydramine. Compared to placebo, psilocybin was associated with increased activation in the medial and lateral prefrontal cortex and left caudate, and decreased activation in the insular cortex, motor cortex, temporal and parietal lobes, and cerebellum. These brain changes were observed in response to both alcohol and emotionally valenced cues. The observed neural patterns suggest enhanced emotional regulation, reduced craving, and potentially improved cognitive control, although the small sample size limits generalizability [48].

While the primary focus of clinical research on psilocybin and substance use has been AUD, early exploratory work has examined its application to other forms of addiction. A single-case report described the experience of a 36-year-old trans woman with daily methamphetamine (MA) use who underwent psilocybin-assisted therapy following inpatient detoxification. The individual reported significant cognitive and emotional changes, including improved mindfulness, better distress tolerance, and abstinence from MA over a three-month follow-up period. Although this case highlights the potential feasibility and safety of PAT for stimulant use disorders, further controlled studies are needed to determine its efficacy in broader populations [69].

#### 5.2.2. Eating Disorders and Body Dysmorphic Disorder (BDD)

Psilocybin-assisted therapy is being explored as a potential intervention for impulse-related and body image disorders such as AN and BDD, which are often resistant to conventional treatment and characterized by persistent cognitive distortions and compulsive behaviors.

A pilot study protocol has been developed to investigate the feasibility, safety, and potential efficacy of psilocybin-assisted therapy in individuals with AN. The ongoing open-label trial at Imperial College London is enrolling 20 adult female participants with a BMI ≥ 15 kg/m^2^. Each participant receives up to three oral doses of psilocybin (maximum 25 mg) over a six-week period, supported by psychological preparation, therapeutic integration, and involvement of care teams and support persons. The primary clinical outcomes include changes in eating disorder psychopathology (as measured by the Eating Disorder Examination) and motivation for recovery (Readiness and Motivation Questionnaire). Neurophysiological assessments, including functional MRI and EEG, will examine changes in brain function and neural plasticity. This study also incorporates public and patient input in its design and aims to inform future randomized controlled trials [27].

For BDD, a recent open-label pilot study evaluated the effects of a single 25 mg dose of psilocybin in 12 adults with moderate-to-severe, non-delusional BDD who had not responded to at least one prior serotonin reuptake inhibitor trial. All participants completed the study, which included psychological support before, during, and after the psilocybin session. Significant reductions in BDD symptoms were observed over the 12-week follow-up period, with improvements noted as early as week 1. The primary outcome, measured by the BDD version of the Yale-Brown Obsessive-Compulsive Scale (BDD-YBOCS), showed a statistically significant and sustained reduction in symptom severity (*p* < 0.001, partial eta squared = 0.54). Secondary measures, including conviction of belief, negative affect, and functional impairment, also improved significantly. By week 12, 58% of participants met response criteria (≥30% reduction in BDD-YBOCS score). No serious adverse events occurred during the study. These preliminary findings support further investigation of psilocybin as a treatment for BDD in larger, controlled trials [33].

### 5.3. Neurological and Emerging Applications

#### 5.3.1. Neurological Disorders and Chronic Pain

Psilocybin is being investigated for its potential therapeutic role in neurological conditions, including chronic cluster headaches (CCH) and chronic neuropathic pain, both of which are challenging to treat and often resistant to conventional therapies.

A small open-label clinical trial evaluated the effects of psilocybin in 10 patients with chronic cluster headaches. Participants received three oral doses of psilocybin (0.14 mg/kg) administered across three consecutive weeks. The first four weeks served as a baseline period, followed by four weeks of post-treatment observation. Headache frequency was reduced by an average of 31% (±31%) from baseline to follow-up (*p*_FWER_ = 0.008). One participant experienced complete remission for 21 weeks. Functional MRI conducted before and after treatment revealed that changes in hypothalamic-diencephalic functional connectivity correlated significantly with reductions in attack frequency (*p*_FWER_ = 0.03, *R* = −0.81). These findings suggest that psilocybin may have a prophylactic effect in CCH and implicate hypothalamic connectivity as a potential mechanism of action. The treatment was reported to be well tolerated, although larger controlled trials are needed to confirm efficacy and safety [28].

In the context of chronic pain, a case series documented the experiences of three individuals who self-administered low (subpsychedelic) doses of psilocybin for chronic neuropathic pain management. Each patient reported long-standing pain unresponsive to standard pharmacological treatments. Across cases, participants achieved notable reductions in pain intensity and reported decreased reliance on traditional analgesics. The analgesic effects were observed without inducing a psychedelic experience and were generally accompanied by minimal cognitive or somatic adverse effects. In one case, the combination of psilocybin and physical exercise enhanced pain relief, and repeated dosing appeared to yield cumulative benefit—suggesting a possible mechanism involving neuroplasticity. Although these anecdotal cases highlight the potential of low-dose psilocybin in managing chronic pain, rigorous clinical trials are needed to establish safety, efficacy, and dosing protocols [70].

#### 5.3.2. Autism, Empathy, and Concussion Recovery

Emerging research has begun exploring the effects of psilocybin on social cognition, neurodevelopmental differences, and post-concussion recovery, with a focus on emotional empathy, autism spectrum disorder (ASD), and symptoms following traumatic brain injury.

A randomized, placebo-controlled clinical trial examined the effects of psilocybin on emotional empathy in individuals with MDD. Fifty-one participants received either a single dose of psilocybin (0.215 mg/kg) or a placebo, within a 4-week psychological support framework. Emotional empathy was measured using the Multifaceted Empathy Test at baseline and at intervals up to two weeks post-treatment. Compared to placebo, psilocybin significantly increased explicit emotional empathy, particularly toward positive emotional stimuli, with effects lasting at least two weeks. These findings suggest that psilocybin may enhance aspects of social cognition in clinical populations and contribute to the psychological mechanisms underlying its antidepressant effects [38].

The role of psilocybin in autism is being explored through the PSILAUT study, a case-control, double-blind, randomized trial investigating serotonergic function in autistic and non-autistic adults. Participants receive low doses of psilocybin (2 mg and 5 mg) or placebo across up to three visits, and neural responses are measured using functional MRI and EEG. The study aims to assess whether serotonin-related brain functions, particularly those mediated by 5-HT_2_A receptors, differ between groups. This is the first experimental study to examine psilocybin’s neurobiological effects in autistic individuals and is expected to inform both the understanding of ASD neurobiology and the design of future clinical trials involving serotonergic compounds in this population [29].

Interest in psilocybin-assisted therapy (PAT) for concussion recovery has also emerged, particularly in athletic populations. A survey-based study assessed the use of psychedelics and attitudes toward PAT among 175 respondents, including 85 athletes and 90 sports staff in Canada and the United States. While regular psychedelic use was low (7.5%) among athletes, psychedelics were the third most commonly used substance in the past year (35.8%). Among athletes, 61.2% indicated a willingness to engage in PAT for post-concussion recovery, and 71.1% of staff reported they would support such use. Path analysis revealed that both psilocybin knowledge and positive attitudes were significant predictors of willingness to engage in or support PAT. These findings suggest a general openness within the sports community toward exploring psilocybin as a treatment for persistent post-concussion symptoms (PPCS), though further clinical research is necessary to evaluate its efficacy and safety in this context [71].

Table 3 categorizes psilocybin research based on its potential therapeutic effects across various disorders, including depression, anxiety, PTSD, OCD, AUD, neurodegenerative diseases, eating disorders, and chronic pain conditions. Clinical trials indicate psilocybin may significantly reduce symptoms of MDD and TRD, with effects lasting weeks to months. Research also suggests potential benefits for anxiety, PTSD, and OCD, though long-term safety and efficacy remain under investigation. Studies on AUD report reductions in alcohol and tobacco consumption, while preclinical evidence suggests possible neuroprotective effects in neurodegenerative conditions. Preliminary trials for eating disorders and chronic pain disorders show promise, but more rigorous clinical research is needed to confirm efficacy and optimize treatment protocols.

## 6. Barriers to Progress in Psilocybin Research

### 6.1. Trial Design and Methodological Limitations

While psilocybin has shown therapeutic promise in MDD and AUD, its efficacy in other conditions remains investigational. Evidence for disorders such as PTSD, AN, concussion recovery, chronic neuropathic pain, and functional dissociative seizures is limited to small-scale, exploratory, or preclinical studies [30,70,71,79,80]. Similarly, no clinical or preclinical studies currently support psilocybin’s use in amyotrophic lateral sclerosis (ALS), and concerns persist regarding psychological risks in neurodegenerative populations [77].

Another major challenge is the small sample size of many psilocybin trials, which restricts statistical power and generalizability. Studies involving OCD, HIV-related shame, AN, and chronic cluster headaches often include too few participants to draw definitive conclusions [26,27,28,45,60,74]. Compounding this issue is a lack of demographic diversity. Most psilocybin studies enroll predominantly White participants, limiting applicability across different racial and ethnic groups. For example, a survey found reduced odds of hypertension in psilocybin users only among White individuals [81]. Greater inclusivity in participant recruitment is essential for equitable clinical translation [13,82].

Further complicating the interpretation of therapeutic outcomes are expectancy effects and psychological suggestibility. Studies indicate that beliefs about psilocybin’s effects—particularly regarding emotional breakthroughs or mystical experiences—can shape outcomes independently of pharmacological action [13,83,84]. This presents a challenge in distinguishing drug-specific effects from contextual influences, especially in the highly suggestible states induced during psilocybin sessions.

In addition, variability in trial design, including inconsistent dosing protocols, inclusion criteria, and outcome measures, hinders cross-study comparison and meta-analysis. Many trials exclude participants with psychiatric comorbidities or those taking concurrent medications, which reduces external validity [15,85]. Moreover, outcome instruments such as the 16-item Quick Inventory of Depressive Symptomatology—Self-Report (QIDS-SR16) may not capture core aspects of psilocybin’s effects, such as ego dissolution or cognitive flexibility, potentially underestimating therapeutic outcomes [17,86].

Many current studies face significant methodological limitations. Trials often feature small, homogeneous samples [25,87], lack active placebo controls [24], or suffer from high expectancy biases [84]. Despite positive trends, some trials fail to meet conventional clinical significance thresholds due to instrument limitations, such as the insensitivity of QIDS-SR-16 to core depressive symptoms [86]. Furthermore, long-term follow-up remains sparse, with some adverse events only emerging weeks post-treatment [17,59]. These gaps underscore the need for more robust, longitudinal study designs incorporating diverse populations, active control conditions, and multidimensional outcome measures that reflect the complexity of psychedelic experiences.

### 6.2. Safety and the Role of the Therapist

Although psilocybin therapy has been associated with rapid and sustained improvements in depression, AUD, and cancer-related distress, long-term safety data remain limited [18,19,23,57,58,88,89,90]. Questions persist regarding the durability of therapeutic effects in chronic or recurrent psychiatric conditions. In particular, certain populations—such as individuals with bipolar disorder, trauma histories, or psychiatric instability—may be at elevated risk for adverse psychological reactions. Documented concerns include mood destabilization, mania, and increased suicidality following treatment [1,9,10,21,59,60,68,72,75,91].

Specialized protocols may be necessary to minimize risk in vulnerable populations. For example, trauma-informed approaches are recommended for individuals with significant trauma histories to avoid reactivation of distressing memories [60], while patients with bipolar disorder may require tailored risk assessment due to the potential for treatment-emergent mania [91].

The therapeutic context also plays a critical role in psilocybin’s efficacy and safety. While models such as ACT and CFT have been proposed, standardized psychotherapeutic protocols remain underdeveloped [46,49,50,51,52,63,92]. Moreover, although the strength of the therapist–patient alliance is known to influence treatment outcomes, few studies systematically evaluate therapist training, session fidelity, or the therapeutic relationship itself [39,53,54].

Developing formal standards for therapist training and integration practices is essential for scaling psilocybin-assisted therapy responsibly and reproducibly.

### 6.3. Translational and Mechanistic Barriers

Despite encouraging clinical outcomes and neuroimaging data, key questions remain regarding psilocybin’s mechanisms of action. Psilocybin is known to alter brain network connectivity, enhance neuroplasticity, and modulate emotional processing, yet the precise pathways through which these effects occur remain incompletely characterized [2,3,12]. For example, changes in the default mode network and serotonergic signaling have been linked to therapeutic effects, but these associations require further validation.

Preclinical studies have shown that psilocybin can reduce alcohol relapse in rodent models and modulate neural activity in reward-related regions such as the nucleus accumbens [35,93,94]. However, translating these findings to clinical populations remains a significant challenge. Bridging this gap will require integrated research approaches that align mechanistic studies with clinical endpoints.

### 6.4. Systemic, Regulatory, and Cultural Challenges

Several external factors continue to hinder the clinical integration of psilocybin. Although biosynthetic production using *Escherichia coli* and enzymatic methods (e.g., PsiK phosphorylation) have improved manufacturing efficiency, cost and scale remain barriers to widespread availability [95,96,97]. Without affordable, pharmaceutical-grade formulations and robust distribution infrastructure, psilocybin therapy may remain limited to academic or private research settings.

Regulatory restrictions also pose major obstacles. In many jurisdictions, psilocybin remains a Schedule I substance, limiting research access and slowing clinical translation. Meanwhile, unregulated psilocybin use in retreat centers and underground therapy settings raises safety and ethical concerns due to the absence of standardized treatment protocols or medical oversight [98].

Cultural and ethical considerations are equally important. Robust informed consent is critical, given psilocybin’s capacity to induce profound changes in self-perception, emotional states, and worldviews [59,61,98]. Moreover, the growing medicalization of psilocybin has raised concerns about cultural appropriation and detachment from Indigenous traditions that have long used psilocybin-containing mushrooms for spiritual and healing purposes. Acknowledging these traditions and ensuring ethical integration into modern frameworks is essential for equitable and respectful use [6].

Table 4 outlines major challenges in psilocybin research and clinical application, including adverse effects, therapist expertise, expectancy effects, microdosing limitations, and ethical concerns. While psilocybin is generally well-tolerated, rare cases of increased suicidality and psychological distress highlight the need for careful patient screening. The lack of standardized therapist training protocols remains a barrier to safe and effective treatment. Expectancy effects and blinding difficulties complicate clinical trial outcomes, while microdosing studies suffer from inconsistent findings and strong placebo effects. Ethical concerns include informed consent, risks of ego dissolution, commercialization issues, and equitable access to treatment. Addressing these challenges through rigorous research, regulatory frameworks, and ethical oversight is crucial for advancing psilocybin’s therapeutic potential.

## 7. Advancing Psilocybin Research: Translation and Integration

### 7.1. Clinical Expansion and Mechanistic Discovery

#### 7.1.1. Expanding Clinical Trials for Diverse Populations and Indications

To confirm the safety and efficacy of psilocybin-assisted therapy, larger multicenter randomized controlled trials (RCTs) are needed across a wider range of clinical conditions. Promising findings have been reported for TRD, AUD, cancer-related psychological distress, and anxiety disorders [5,10,21,67,88,89,90]. However, the evidence base remains limited for several emerging indications, including AN, BDD, autism spectrum disorder (ASD), concussion recovery, and chronic neuropathic pain [29,33,71,80,82,102]. These conditions require additional evaluation through rigorously designed, controlled clinical trials to determine therapeutic efficacy and establish safety profiles.

A critical issue in current psilocybin research is the underrepresentation of diverse populations. Many clinical trials to date have enrolled primarily White participants, limiting the generalizability of findings and potentially overlooking differences in treatment response across racial, ethnic, cultural, and socioeconomic groups [13,81,82]. Expanding recruitment strategies to include more diverse populations is essential to ensure that psilocybin-assisted therapy can be equitably applied in real-world clinical settings and that outcome disparities are appropriately addressed.

#### 7.1.2. Long-Term Follow-Up and Monitoring

While psilocybin has demonstrated rapid symptom reductions in depression, AUD, PTSD, and cancer-related anxiety, limited data are available regarding the long-term durability of these effects [12,18,67,88,101]. Future clinical trials should incorporate extended follow-up periods to assess the persistence of therapeutic benefits, the likelihood of relapse, and the need for maintenance interventions [18,23,58].

In parallel, ongoing safety monitoring is essential—particularly for individuals with elevated clinical risk profiles. This includes patients with histories of trauma, eating disorders, bipolar disorder, or co-occurring medical and psychiatric conditions [4,9,13,59,82,91]. Structured follow-up protocols should be developed to track adverse events over time, support psychological integration, and identify early indicators of relapse or destabilization. These monitoring systems will be crucial for transitioning psilocybin from investigational use to routine clinical practice.

#### 7.1.3. Advancing Mechanistic Understanding

A central research objective is to elucidate the neurobiological mechanisms through which psilocybin exerts its therapeutic effects. Preclinical and neuroimaging studies have consistently shown that psilocybin enhances neuroplasticity, modulates serotonergic activity, and alters brain network connectivity. These mechanisms are thought to contribute to sustained improvements in mood and emotional regulation in disorders such as depression and addiction [2,29,31,36,103].

Specific brain regions have emerged as potential mediators of psilocybin’s clinical effects. For instance, in studies involving alcohol use, psilocybin administration reduced self-administration behavior in rodents and was associated with modulation of the nucleus accumbens, a region implicated in reward processing and addiction [35]. At the systems level, functional MRI studies in both depressed and alcohol-dependent populations have demonstrated increased global brain network integration and decreased connectivity within the default mode network (DMN)—a network often associated with rumination and self-referential thought patterns [2,41].

Although these findings are promising, further investigation is needed to clarify the roles of specific regions such as the prefrontal cortex and amygdala, particularly in relation to emotional regulation and mood stabilization [29,31]. Additionally, the identification of reliable neurobiological markers for predicting treatment response remains an open area of research. Clinical trials such as the EMBRACE study are currently exploring psilocybin’s effects on brain function using advanced neuroimaging techniques; however, conclusive evidence linking specific biomarkers to therapeutic outcomes is still lacking and warrants continued exploration [41,104].

### 7.2. Therapeutic Model Development and Optimization

#### 7.2.1. Refining and Standardizing Therapeutic Models

The development of standardized psychotherapeutic models is essential for ensuring consistency, safety, and efficacy in psilocybin-assisted therapy across clinical and research settings. Existing models such as ACT and CFT have demonstrated the potential to enhance core therapeutic processes, including emotional regulation, psychological flexibility, and self-compassion [49,50,51]. Tailoring these models to meet the specific needs of patient populations—such as individuals with cancer—may further improve treatment outcomes [30,67,101]. However, their applicability to other groups, such as military veterans and individuals with complex trauma histories, requires additional investigation.

Beyond formal psychotherapeutic models, the broader therapeutic context—including preparation, integration, and rapport-building—is considered a central component of treatment success. Research has shown that non-pharmacological elements such as music, structured rituals, and personalized therapeutic frameworks can enhance the subjective and clinical outcomes of psilocybin experiences [39,46,100].

Efforts to standardize these components have led to the development of structured models like the Compass Psychological Support Model (CPSM), as well as the establishment of consensus-based psilocybin service measures. These frameworks provide guidance on patient preparation, session structure, therapist training, and safety monitoring. Continued refinement of these therapeutic models is necessary to support replicability across trials and facilitate the transition from research environments to regulated clinical practice [52,63].

#### 7.2.2. Developing Comprehensive Safety Guidelines

Establishing comprehensive safety protocols remains a key priority in the development of psilocybin-assisted therapy. Cardiovascular safety data suggest that psilocybin, at therapeutic doses, has minimal impact on QTc intervals, indicating a low risk of cardiac arrhythmia [1,7,8,105]. However, safety in individuals with psychiatric vulnerabilities—including those with trauma histories, bipolar disorder, or concurrent psychiatric diagnoses—requires further investigation and specific risk mitigation strategies.

Preliminary clinical data indicate that psilocybin may be safely co-administered with SSRIs. A phase II study involving patients with TRD found that concurrent SSRI use did not result in serious adverse events and was associated with significant improvements in depressive symptoms [12,64]. Nonetheless, larger-scale studies are necessary to confirm the safety and efficacy of combined pharmacological approaches.

Informed consent procedures must address the full range of potential psychological effects, including ego dissolution, transient anxiety, and the emergence of distressing emotional material. These effects, while often therapeutic when properly supported, require comprehensive patient education to ensure psychological preparedness and minimize risk [59,61].

#### 7.2.3. Optimizing Dosing Strategies

Refining psilocybin dosing protocols is an important area of ongoing research. Current evidence suggests that body weight does not significantly influence subjective effects or treatment outcomes, indicating that weight-adjusted dosing may not be necessary in most cases [34]. However, the potential roles of other individual factors—such as sex, metabolic rate, and prior psychedelic experience—remain understudied and should be incorporated into future pharmacokinetic (PK) and pharmacodynamic (PD) analyses.

Although existing trials have demonstrated psilocybin’s efficacy in reducing depressive symptoms, few have directly compared its therapeutic value to that of other interventions, such as SSRIs or ketamine. As such, psilocybin’s relative position within psychiatric treatment hierarchies remains undefined [13,20]. Moreover, while some studies have employed fixed-dose designs, the comparative effectiveness of single high-dose versus multiple moderate-dose approaches remains unexplored in large-scale trials. Adaptive dosing strategies, which tailor the dose based on individual response, have yet to be formally tested in psilocybin research and represent an important direction for future clinical optimization [20].

### 7.3. Innovation, Industry, and Holistic Care

#### 7.3.1. Exploring Combination Therapies and Novel Applications

Psilocybin’s therapeutic potential may be enhanced when explored alongside other agents or in novel clinical applications. Preliminary research has considered the possible synergistic use of psilocybin and ketamine, given their distinct pharmacological mechanisms. Psilocybin primarily targets serotonin 5-HT_2_A receptors, while ketamine acts on NMDA receptors, suggesting the potential for complementary effects in psychiatric treatment. However, direct clinical evidence supporting their combined use remains limited [12].

Other potential adjunctive strategies, such as combining psilocybin with mindfulness-based interventions or conventional antidepressants, have been proposed in theory but lack empirical validation. Further research is required to assess the safety and efficacy of these integrated approaches [50].

Emerging therapeutic areas include psilocybin-assisted therapy (PAT) for concussion recovery. A survey of 175 athletes and sports staff found that 60% of athletes were open to trying PAT for concussion-related symptoms, and 71% of staff supported its potential use. However, current evidence remains survey-based, and there are no clinical trials supporting psilocybin for functional neurological disorders or HIV-related stigma, indicating a need for further investigation in these areas [30,71,102].

Evidence for psilocybin’s effect on emotional empathy has been demonstrated in individuals with MDD, where a randomized controlled trial reported increased empathy toward positive emotional stimuli lasting at least two weeks post-treatment. Additionally, preclinical studies suggest that psilocybin may support cognitive flexibility, a function relevant in AN, although direct clinical evidence in this context is currently limited. In autism spectrum disorder (ASD), ongoing trials such as the PSILAUT protocol are investigating psilocybin’s effects on serotonergic systems, but further clinical validation is needed [29,33,38].

#### 7.3.2. Industrial Optimization and Pharmaceutical Development

To support clinical and pharmaceutical scalability, ongoing innovation in psilocybin production is essential. Biosynthetic methods—such as recombinant psilocybin production in *Escherichia coli*—have demonstrated production titers up to 1.16 g/L, offering a promising platform for large-scale synthesis [95]. Additionally, hybrid synthetic-biocatalytic approaches using the enzyme PsiK have enabled efficient phosphorylation of psilocin, streamlining production and reducing reliance on chemical synthesis [96].

Further development in crystallization techniques and purification processes may enhance the consistency and stability of clinical-grade psilocybin formulations [106,107]. Pharmaceutical innovation has also extended to the creation of novel psilocybin analogs. Modifications such as methylation or alteration of the tryptamine backbone are being investigated to target specific receptor subtypes or modulate pharmacokinetics, including the onset and duration of effects [11,107]. These efforts aim to optimize the therapeutic profile and improve clinical applicability across different psychiatric conditions [108].

#### 7.3.3. Addressing Ethical, Regulatory, and Societal Barriers

Despite growing clinical interest, regulatory challenges continue to hinder the widespread adoption of psilocybin-assisted therapy. An analysis of 134 clinical trials identified inconsistencies in research design and fragmentation across studies as barriers to regulatory progress [85]. Although psilocybin has received “breakthrough therapy” designation from the U.S. Food and Drug Administration (FDA) for TRD, there is no direct evidence from cited sources identifying this designation—or psilocybin’s Schedule I classification—as the primary impediments to clinical adoption [56,85].

Public perception of psilocybin therapy is increasingly favorable. In a Norwegian population survey (N = 1,078), 51% of respondents indicated they would consider using psilocybin for medical purposes, despite only 8% reporting prior use [62]. However, there is currently no direct evidence supporting the effectiveness of policy collaboration initiatives or stigma reduction campaigns related to psilocybin [81,85].

Ethical concerns have also been raised regarding the disconnection between psilocybin’s medicalization and its traditional use in Indigenous spiritual practices. A bibliometric analysis comparing clinical psilocybin and ayahuasca research found that psilocybin trials predominantly emphasize Western clinical contexts, while ayahuasca research tends to retain cultural and spiritual framing [6]. These findings underscore the importance of culturally sensitive approaches and ethical stewardship as psilocybin continues to enter regulated medical systems.

#### 7.3.4. Holistic and Spiritual Care

Psilocybin’s potential to address existential and spiritual dimensions of distress has been most clearly demonstrated in individuals facing life-threatening illnesses. In cancer patients, psilocybin-assisted therapy has led to reductions in existential despair, including loss of meaning and suicidal ideation. A randomized controlled trial reported that these improvements persisted for up to 4.5 years in some participants [58].

While the therapeutic integration of spiritual experiences has been reported in AUD treatment, where patients described encounters with divine entities and religious figures, no direct evidence from the cited studies supports the use of psilocybin in addiction recovery as a spiritually integrated intervention [57,109]. Furthermore, although concepts such as guided rituals and meaning-centered psychotherapy are often discussed in the theoretical literature, they are not explicitly studied or referenced in the clinical trials cited [46,68]. Additional research is required to evaluate the role of structured spiritual care frameworks in psilocybin-assisted therapy and their potential contribution to treatment outcomes.

To support the translation of these insights into actionable steps, Table 5 outlines key implementation strategies aligned with the objectives presented in this section. Each strategy is paired with supporting references to guide future research, clinical development, and policy efforts. This table serves as a practical roadmap for advancing psilocybin research through integrated, multidisciplinary approaches.

## 8. Conclusions

Psilocybin-assisted therapy represents a paradigm shift in psychiatric and neurological treatment, offering a potential breakthrough for conditions resistant to conventional interventions. While clinical trials demonstrate its efficacy in depression, anxiety, alcohol use disorders, and existential distress, critical challenges must be addressed before widespread clinical adoption. Methodological improvements, including larger, more diverse study populations and long-term safety assessments, are essential. Standardized therapeutic models and training for facilitators must be developed to ensure consistency and efficacy. Advances in regulatory frameworks, biosynthetic production, and interdisciplinary collaboration will be crucial for transitioning psilocybin from experimental research to mainstream medicine. Equitable access and ethical considerations, particularly regarding indigenous knowledge and cultural sensitivity, must be prioritized. Future research must refine dosing strategies, elucidate neurobiological mechanisms, and establish guidelines for safe and effective integration. By addressing these challenges, psilocybin has the potential to transform mental healthcare and redefine therapeutic approaches for psychiatric and neurological disorders.

## Figures and Tables

**Table 1:** Overview of psilocybin research categories.

Category	Description	Key Findings	Example Studies
Systematic Reviews	Comprehensive analyses of multiple studies on psilocybin’s efficacy, safety, and mechanisms.	Psilocybin-assisted therapy may reduce symptoms of depression, anxiety, and AUD. Current findings are limited due to small sample sizes, expectancy effects, inconsistent dosing, and legal barriers. Mild to moderate adverse effects are commonly reported. Rare but serious adverse events have occurred and require further investigation. Larger, well-controlled trials are necessary before clinical adoption.	[4,9,10,12,13,14,15,16,17]
Clinical Trials	Controlled studies testing psilocybin’s therapeutic effects, often comparing it to placebos, active comparators, or standard treatments.	Randomized controlled trials show significant symptom reduction in depression and AUD. Some studies suggest efficacy comparable to antidepressants. Therapeutic effects vary between individuals. Expectancy effects may influence outcomes. Short-term adverse effects are frequently observed. Long-term safety data remain insufficient. More trials are needed to determine optimal treatment protocols.	[5,18,19,20,21,22,23,24,25]
Special Population Investigations	Studies on psilocybin’s effects in specific groups, including cancer patients, veterans, and individuals with psychiatric or neurological conditions.	May reduce cancer-related depression, Post-Traumatic Stress Disorder (PTSD) symptoms in veterans, and distress in treatment-resistant psychiatric conditions. Preliminary research indicates possible benefits for anorexia nervosa (AN) and chronic cluster headaches. Safety concerns are notable in vulnerable populations. Individualized protocols are recommended. Large-scale, controlled trials are necessary for validation.	[26,27,28,29,30,31,32,33]

**Table 2:** Summary of psilocybin’s pharmacokinetic, pharmacodynamic, and neurobiological effects.

Category	Description	Key Findings	Example Studies
Pharmacokinetics	Studies on how psilocybin is absorbed, distributed, metabolized, and excreted, with a focus on half-life, bioavailability, and clearance.	Psilocybin is rapidly metabolized into psilocin, which exhibits dose-proportional kinetics. Psilocin reaches peak plasma concentration approximately 2 h after dosing. It has an elimination half-life of about 3 h. Minimal renal clearance indicates no need for dose adjustment in cases of mild-to-moderate renal impairment. Linear pharmacokinetics across a range of doses provide reliable reference data for clinical trial design.	[1,34]
Pharmacodynamics	Research on psilocybin’s interaction with serotonin receptors and its effects on cognition, mood, and perception.	Psilocybin primarily functions as a partial agonist at the 5-HT2A receptor, modulating serotonin pathways. This activity influences perception, mood, and cognition. Antagonism of 5-HT2A receptors blocks some of psilocybin’s effects. It also interacts with dopamine D2 receptors and modulates glutamate signaling. These effects contribute to its behavioral and therapeutic potential in psychiatric disorders.	[35,47]
Neurobiological Effects	Neuroimaging and molecular studies examining psilocybin’s effects on brain connectivity, network integration, and potential neuroplasticity.	fMRI studies show reduced activity in the default mode network (DMN) and increased global brain integration. These changes are associated with enhanced cognitive flexibility and emotional processing. Altered connectivity is observed in brain networks related to emotional regulation and addiction. Preclinical studies suggest psilocybin affects glutamate signaling and may promote neuroplasticity. Long-term structural effects remain uncertain and require further research.	[2,3,41,42,48]

**Table 3:** Psilocybin Research by Disorder.

Disorder	Description	Key Findings	Example Studies
Major Depressive Disorder (MDD)	Clinical trials show psilocybin reduces depression severity, with effects lasting from weeks to months. Long-term efficacy and safety need further study.	Psilocybin significantly reduces depression severity, with effects lasting from weeks to months. Some studies indicate efficacy comparable to traditional antidepressants. Individual response variability remains a major area of ongoing research.	[10,18,19,20,25]
Treatment-Resistant Depression (TRD)	Psilocybin trials show significant symptom relief, with some patients achieving remission.	Symptom reductions can be sustained even in patients with prior antidepressant use. Repeated dosing and integration with psychotherapy show potential benefits. Individualized treatment approaches are important. Response variability in complex cases and concerns over long-term safety require further investigation.	[23,66,72]
Anxiety Disorders	Studies suggest that psilocybin-assisted therapy may alleviate anxiety, particularly in cases of existential distress, such as terminally ill patients and long-term survivors of serious illnesses.	Reduces anxiety-related distress, particularly in terminally ill patients. Group therapy settings may enhance therapeutic outcomes. Current findings are mainly limited to existential distress. Limited evidence for generalized anxiety disorder. Long-term safety and efficacy require further clinical validation.	[26,32,73]
Post-Traumatic Stress Disorder (PTSD)	Preliminary research suggests psilocybin-assisted therapy may help reduce PTSD symptoms and improve emotional processing, particularly in military veterans with treatment-resistant PTSD.	Early research suggests reductions in PTSD symptoms, especially in veterans with treatment-resistant PTSD. Safety concerns persist for trauma-exposed populations. Some individuals report worsened distress. Controlled trials are needed to confirm efficacy and assess long-term outcomes.	[30]
Obsessive-Compulsive Disorder (OCD)	Clinical trials are investigating psilocybin’s potential to reduce compulsive behaviors and intrusive thoughts in individuals with treatment-resistant OCD.	Preliminary studies indicate temporary reductions in OCD symptoms with single-dose psilocybin. Controlled trials are ongoing to assess long-term efficacy, neurobiological mechanisms, and optimal dosing. No standardized treatment guidelines exist yet.	[45,74]
Alcohol Use Disorders (SUDs)	Psilocybin-assisted therapy has been linked to reductions in alcohol and substance use, with stronger psychedelic experiences correlating with better treatment outcomes.	Clinical trials show significant reductions in alcohol use, with effects lasting up to 36 weeks. Personality changes, such as decreased neuroticism and impulsivity, may aid treatment success. Continued studies are evaluating efficacy, safety, and dosing protocols.	[5,44,56,75,76]
Neurodegenerative Disorders	No clinical evidence currently supports psilocybin as a treatment for neurodegenerative diseases such as ALS or Parkinson’s disease.	No clinical evidence currently supports psilocybin as a treatment for ALS or Parkinson’s disease. Theoretical neuroprotective mechanisms remain speculative. Psychological risks in vulnerable populations necessitate caution. Further research is needed.	[77]
Eating Disorders	Psilocybin is being investigated as a potential therapy for eating disorders, including AN and BDD.	Early trials indicate reductions in maladaptive thoughts and improved psychological flexibility in BDD. Ongoing research is exploring effects on eating disorder pathology and neurophysiology. Clinical validation is needed to ensure safety and efficacy.	[27,33]
Cluster Headaches & Chronic Pain	Preliminary research suggests psilocybin may reduce the frequency and severity of cluster headaches, though rigorous clinical trials are needed to confirm efficacy.	Preliminary findings suggest reduced frequency and severity of cluster headaches. Open-label studies report symptom relief. Small sample sizes and methodological limitations hinder definitive conclusions. Additional trials are needed to evaluate efficacy, safety, and dosing strategies.	[28,78]

**Table 4:** Key challenges in psilocybin research and application.

Challenge	Description	Key Considerations	Example Studies
Adverse Effects	Psilocybin is generally well-tolerated, but mild side effects are common, and rare serious effects occur in vulnerable individuals.	Most side effects—such as nausea, headaches, and anxiety—are mild and temporary. Rare instances of increased suicidality and prolonged psychological distress have been reported. These findings underscore the importance of thorough participant screening and ongoing monitoring during treatment.	[4,7,9,14]
Therapist Expertise	Effective therapy requires trained facilitators, but standardized training programs are still in development.	Training programs are essential to promote participant safety, therapeutic consistency, and best clinical practices. Current efforts aim to establish standardized competencies, proper supervision, and adherence to ethical guidelines.	[39,54,63,99]
Expectancy Effects	Patient expectations and placebo effects strongly influence treatment outcomes, complicating assessments of psilocybin’s true effects.	Expectancy effects may inflate perceived therapeutic benefits. Improved blinding methods and robust study designs are necessary to isolate true pharmacological effects. Blinding difficulties remain a significant limitation in current research.	[83,84]
Microdosing Limitations	Research on microdosing is inconclusive, with mixed findings on mood, cognition, and creativity.	Some reported benefits may result more from participants’ expectations than from the drug’s pharmacological action. More rigorously controlled trials are needed to accurately assess psilocybin’s true therapeutic value.	[16]
Ethical Concerns	Issues include informed consent, psychological risks, commercialization, and equitable access to psilocybin therapy.	Ongoing ethical debates concern responsible treatment frameworks, potential commercialization risks, and equitable access to care. Further research is required to understand the long-term psychological impacts of psilocybin use.	[61,98,100,101]

**Table 5:** Implementation strategies for advancing psilocybin research.

Objective	Implementation Strategy	Supporting References
Expand Clinical Trials	Initiate large-scale multicenter RCTs for under-researched conditions (e.g., AN, BDD, ASD, concussion, chronic pain). Collaborate with specialized centers to ensure rigor.	[29,33,71,80,82,102]
Increase Population Diversity	Incorporate inclusive recruitment frameworks, including outreach to underserved communities, translation services, and engagement with local health organizations.	[13,81,82]
Ensure Long-Term Monitoring	Implement 6–24-month follow-up protocols. Include psychological integration, relapse tracking, and safety monitoring systems, especially for high-risk patients.	[12,18,23,58,67,88,101]
Advance Mechanistic Research	Expand fMRI/PET studies in trials; target biomarkers; explore brain regions (e.g., PFC, amygdala); support translational models and trials like EMBRACE.	[2,29,31,35,36,41,103,104]
Standardize Therapeutic Models	Apply structured models like ACT, CFT, and CPSM; ensure therapist training; standardize preparation and integration components.	[30,49,50,51,52,63,67,100,101]
Establish Safety Guidelines	Formalize psychiatric risk screening; expand SSRI interaction trials; improve informed consent and emergency protocols for adverse effects.	[1,4,7,8,9,12,13,59,64,82,91,105]
Optimize Dosing	Compare fixed vs. adaptive dosing in trials; investigate influences of sex, prior psychedelic experience, and metabolism; enhance PK/PD research.	[13,20,34]
Innovate Therapeutically	Pilot combination therapies (e.g., with ketamine or mindfulness); conduct exploratory trials in concussion, ASD, and AN.	[12,29,30,33,38,50,71,102]
Improve Manufacturing	Advance biosynthetic/enzymatic production (e.g., PsiK enzyme); develop improved purification and formulation methods for scalable clinical use.	[11,95,96,106,107,108]
Address Regulatory and Ethical Issues	Establish policy and ethics coalitions; streamline protocols; integrate frameworks that honor Indigenous traditions and cultural sensitivity.	[6,56,62,81,85]
Enhance Holistic/Spiritual Care	Test structured spiritual care interventions; evaluate therapeutic value of existential meaning-making in serious illness and addiction contexts.	[46,57,58,68,109]

## Data Availability

No new data were created or analyzed in this study. Data sharing is not applicable.
